# Biopsychosocial Assessment of Pain with Thermal Imaging of Emotional Facial Expression in Breast Cancer Survivors

**DOI:** 10.3390/medicines5020030

**Published:** 2018-03-30

**Authors:** David Alberto Rodríguez Medina, Benjamín Domínguez Trejo, Patricia Cortés Esteban, Irving Armando Cruz Albarrán, Luis Alberto Morales Hernández, Gerardo Leija Alva

**Affiliations:** 1 Division of Research and Postgraduate Studies, Faculty of Psychology, Universidad Nacional Autónoma de México, Mexico City 04510, Mexico; benjamin@unam.mx; 2 National Medical Center 20 de Noviembre, Instituto de Seguridad y Servicios Sociales de los Trabajadores del Estado, Mexico City 03229, Mexico; dra.pcortes@gmail.com; 3 CA Mechatronics, Faculty of Engineering, Universidad Autónoma de Querétaro, San Juan del Río City 76807, México; irving_cruz93@hotmail.com (I.A.C.A.); luis_morah@yahoo.com (L.A.M.H.); 4 Interdisciplinary Center of Health Sciences, Instituto Politécnico Nacional, Unidad Santo Tomás, Mexico City 11340, Mexico; gelealipn@yahoo.com.mx

**Keywords:** breast cancer, facial expression, IL-6, thermography, alexithymia, social support

## Abstract

**Background**: Recent research has evaluated psychological and biological characteristics associated with pain in survivors of breast cancer (BC). Few studies consider their relationship with inflammatory activity. Voluntary facial expressions modify the autonomic activity and this may be useful in the hospital environment for clinical biopsychosocial assessment of pain. **Methods:** This research compared a BC survivors group under integral treatment (Oncology, Psychology, Nutrition) with a control group to assess the intensity of pain, behavioral interference, anxiety, depression, temperament-expression, anger control, social isolation, emotional regulation, and alexithymia and inflammatory activity, with salivary interleukin 6 (IL-6). Then, a psychophysiological evaluation through repeated measures of facial infrared thermal imaging (IRT) and hands in baseline—positive facial expression (joy)—negative facial expression (pain)—relaxation (diaphragmatic breathing). **Results:** The results showed changes in the IRT (*p* < 0.05) during the execution of facial expressions in the chin, perinasal, periorbital, frontal, nose, and fingers areas in both groups. No differences were found in the IL-6 level among the aforementioned groups, but an association with baseline nasal temperature (*p* < 0.001) was observable. The BC group had higher alexithymia score (*p* < 0.01) but lower social isolation (*p* < 0.05), in comparison to the control group. **Conclusions:** In the low- and medium-concentration groups of IL-6, the psychophysiological intervention proposed in this study has a greater effect than on the high concentration group of IL-6. This will be considered in the design of psychological and psychosocial interventions for the treatment of pain.

## 1. Introduction

Pain is a common symptom in cancer survivors [1]. Pain rating guides for these patients have recently advanced [2]. However, there are few studies evaluating the biopsychosocial processes of pain in cancer patients [1,3,4,5,6]. The evaluation of emotional aspects in the diagnosis of chronic diseases can contribute to optimizing clinical care, adherence to treatment, and promotion of changes in disease behavior. Depending on individual characteristics and oncological medical treatment, breast cancer (BC) represents a challenge for clinical management. Those who suffer from this disease go through a series of physiological, behavioral, cognitive, and affective changes that facilitate or impede the proper management of the disease [7]. Evidence suggests that patients who have a greater institutional support (such as medications, support groups, and so forth), social backing (family and friends), and adaptive emotional regulation resources (cognitive reevaluation, for example) have better medical, affective, and behavioral prognosis management of the BC, compared to those who lack these supports or emotional regulation strategies [8].

That being said, and despite the fact that the social interactions have a potential weight in the reduction of stress hormones and emotional health [9], it is worth mentioning that there is almost no research about the inflammatory response based on the emotional state. However, interleukin 6 (IL-6), a cytokine activated by the chronic stress inflammation process [10,11], has shown its importance when it comes to observing the emotional changes and the level of pain [2]. Its involvement in the regulation of the immune-nervous system has been validated by numerous researchers and shows a high sensitivity to variations in mood [12,13] and social support [14].

In addition to this, the autonomic activity that accompanies the specific emotional changes have been evaluated with thermography, a non-invasive and non-obstructive technological tool [15], that allows a real-time measurement of the changes in temperature, associated with blood supply directly innervated by changes in sympathetic and parasympathetic activity [16,17]. This technique has been documented in a literature review where it has been used to show thermal changes in various regions of observable interest (ROI), such as hands and face, according to different emotional states [18]. In fact, it is relevant to indicate that only in a few investigations has temperature been measured during the performance of voluntary facial expression to assess changes in temperature that accompany the muscle activity in different facial expressions [19,20,21]. Levenson, Ekman, and Friesen [22] designed an experiment with theatre actors to evaluate the autonomic changes associated with different facial expressions. More recently, Kraft and Pressman [23] conducted an intriguing investigation in which they detected and observed a clinical cardiovascular effective change associated with the stress response which was produced by manipulation of facial expression. The monitoring of these autonomic changes could be clinically beneficial for patients who experience some difficulties related to emotional expression and its regulation [24]. This difficulty has been termed alexithymia, and it has been well documented that women survivors of BC who suffer from chronic pain have this condition and it works as a predictor of postoperative pain of more than 12 months [25]. Owing to the fact that research reports indicate that these patients present a decrease in their levels of physical, mental, and social quality of life [7], our study has two objectives. The first one is to explore the relationship between the psychosocial characteristics and psychobiological characteristics of a group of BC survivors compared with a control group. This approach is intended to devise a biopsychosocial profile about the physical and emotional state, and quality of life. On the other hand, the second objective is proposed to investigate the autonomic thermal changes associated with affective states of facial expression: baseline, positive (joy), negative (pain), and relaxation. All the measures were compared with a control group to evaluate not only the clinical variances, but also the statistical differences.

As a result, it is worth stating that we had three hypotheses: (1) the biopsychosocial profile in both groups could be different; (2) the voluntary emotional facial expressions generated thermal facial changes associated with sympathetic and parasympathetic activity; (3) there is a relationship between IL-6 and temperature of the facial ROI.

## 2. Method

### Participants

We requested the voluntary participation of two different groups. The former was a group of breast cancer survivors (*N* = 15, *M* = 56.12 years of age, mean survival time = 10.18 years, schooling *M* = 12 years) who were treated at National Medical Center 20 de Noviembre. The latter was a healthy control group (*N* = 18) with a mean age of 41 years, average schooling ≥ 12 years, without pain. In both groups, the inclusion criteria were being able to read and write. The exclusion criteria were fasting less than 3 h prior to data collection (IL-6 salivary sample). This study was approved by the ethics committees of the Oncology Section National Medical Center 20 de Noviembre, and Faculty of Psychology of the National Autonomous University of Mexico. All subjects provided written informed consent for participation in the study.

## 3. Measurement

Because the group of patients did not have a psychological evaluation, we decided to create a biopsychosocial profile, which was made not only by evaluating their affective, cognitive, and perceived pain characteristics, but also by taking into account the inflammatory activity (IL-6) and the autonomic activity of facial temperature.

### 3.1. Psychometric Measures

Short Form of Questioning Mc Hill Pain (SF-MPQ) [26]. The short version of the McQueen Pain Questionnaire (MPQ), validated in the Mexican population [27], consists of 15 pain descriptors, 11 of which are sensorial, while the remaining four assess the affective aspect. As a result, this questionnaire has been regarded as an excellent indicator of pain, with psychometric properties (α = 0.73–0.89) [28]. In fact, it has been used for carrying out some investigations connected with cancer patients [29].

Brief Pain Inventory (BPI). This questionnaire assesses pain intensity (maximum, minimum, average, and current), as well as the degree of interference in daily activities of the patient (general activity, work, walking, mood, interpersonal relationships, sleep quality, and fun) through a numerical scale (0—nothing, at 10—extreme). This questionnaire is known for its adequate psychometric properties. In addition, it is translated into Spanish and it is commonly used in the oncological population worldwide, including Mexico [30]. In fact, in 2009, Mexico’s Ministry of Health implemented its version for the diabetic population. It showed a level of pain and behavioral interference of 0–3.3, a moderate level of 3.31–6.6, and a high pain level of 6.7.

Hospital Anxiety and Depression Scale (HADS). This questionnaire measures the psychological distress of anxiety and depression. As for the Mexican version of this test, it was proven an excellent tool in patients with cancer and it consists of 12 items (6 for anxiety and 6 for depression) on a Likert type scale of 0 to 3. Its utility among the Mexican population with cancer has estimated clinical cut points for both factors: 0 to 5 points, without; 6 to 8 points, slight; 9 to 11 points, moderate; and 12 onwards, severe [31].

Primary Care PTSD Screen (PC-PTSD) [32]. It consists of four items with dichotomous responses (yes or no) about the symptomatology of post-traumatic stress disorder. Consequently, it indicates the presence of this disorder with either three or four positive answers. Finally, it is relevant to mention that Tauben [33] proposed this questionnaire for the evaluation of a stepped model of chronic pain.

Expression feature of anger trait—state version 2 (STAXI-2). Validated for the Mexican population [34], it contains 49 reagents distributed in 8 factors with exceptional psychometric properties. For this investigation, four of these factors were used (State, α = 0.853, Temperament, α = 0.860, Internal Control of Ira α = 0.846 and External Control of Ira, α = 0.809) of a total of 27 reagents.

The response options are a Likert-type scale: almost never = 0; Sometimes = 1, often = 2, and almost always = 3.

Toronto Alexithymia Scale (TAS-10). This is the most widely used scale for the evaluation of alexithymia, hence it has been changed on several occasions. The original version [35] consisted of 20 reagents grouped into three factors. However, a short version validated for Mexico’s population [36], includes only two factors: Difficulty expressing feelings and Difficulty in identifying Feelings, that is used in the clinical population with vulnerability to chronic pain [37]. There are six response options, ranging from 0 = Strongly Disagree, to 5 = Strongly Agree. The final score is divided between the number of reagents and the quotient; therefore, it allows us to estimate the level of alexithymia with cut-off points not only for the total, but also for factor 1 = Difficulty of identifying own feelings and emotions; and for factor 2 = Difficulty expressing feelings and emotions to others.

Emotional Questionnaire Regulation (EQR). The Spanish version of the emotional regulation questionnaire was used [38], and it demonstrated a respectable reliability and validity levels. It consists of 10 Likert-type reagents ranging from 1 = Strongly Disagree to 7 = Strongly Agree, and it is divided into 2 factors: Cognitive Reevaluation (6 items) and Emotional Suppression (4 items).

UCLA Loneliness Scale (Version 3) [39]. Evaluates the degree of social isolation. It has good psychometric properties. It consists of answering 20 items on a Likert scale ranging from never (1) to always (4). The higher the score, the greater the degree of social isolation.

### 3.2. Psychobiological Measures

**Figure 1:**
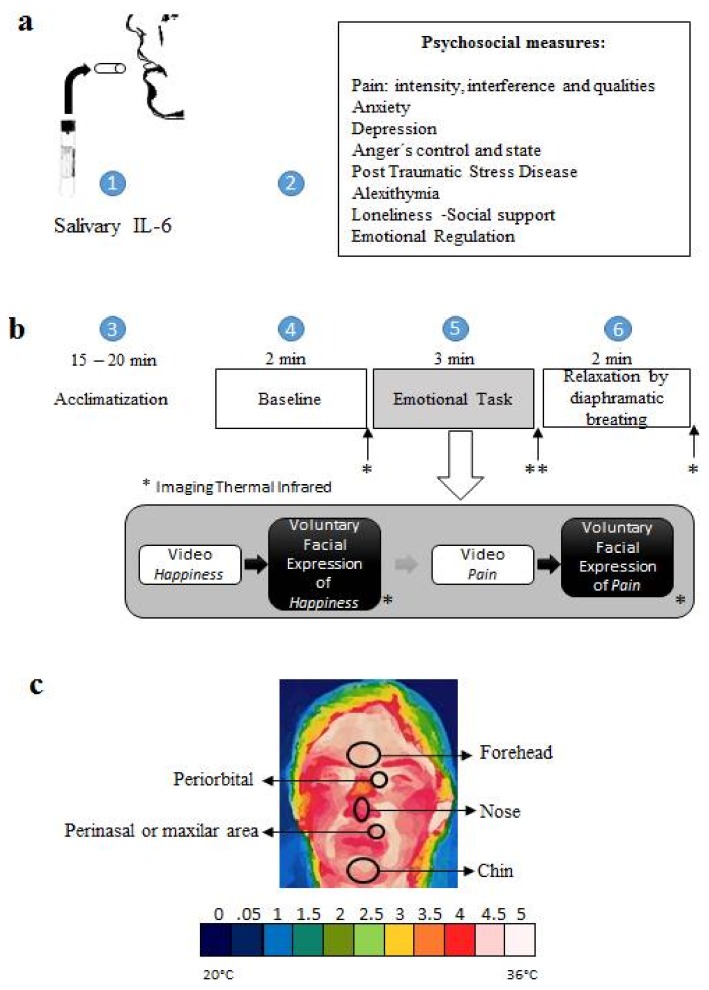
Experimental approaches: (**a**) Salivary IL-6 and Psychological assessment; (**b**) Schematics illustrating the experimental design used in this study with iTF: (1) Baseline (neutral picture). (2) Emotional Task was paradigm Muscle by Muscle to Happy facial expression and Pain facial expression. (3) Relaxation by diaphragmatic breathing; (**c**) Temperature level score and each facial ROI.

*Interleukin 6 salivary (IL-6)*. The salivette kit for IL-6 was used to collect a non-invasive salivary sample of the proinflammatory cytokine (Figure 1a). The salivary samples were obtained according to the instructions of the manufacturer Sarstedt, Newton, NC, and centrifuged at 3000 rpm for 15 min. Serum was stored at −70 °C in pyrogen-free plastic tubes until the time of the test. The concentration of IL-6 was determined by an ELISA kit based on the instructions of the manufacturer Salimetrics State College PA, at a dilution of 1:5. IL-6 concentrations were determined by using a luminescence counter at 450 nm with a secondary filter of 620 to 630 nm. The standard adjustment curve was then performed to obtain the IL-6 concentrations of each of the study participants.

*Infrared Thermal Imaging*. We use a thermographic camera, FLIR A310, FLIR SYSTEMS Inc. (Nashua, NH, USA) which was positioned at a distance of 1.5 m from the tip of each patient’s nose, in a room with controlled ambient temperature of 22 ± 2 °C. Frequency of 30 Hz, infrared λ = 7.5 a 13 μm. We proceeded according to the specifications proposed by Sillero, Fernández, Arnaiz, & Bouzas [40]. The thermographic recording was performed three times: (A) baseline (2 min); (B) emotional task (voluntary emotional facial reproduction, 10 min.); (C) recovery guided by diaphragmatic breathing (2 min). Thermographic images were recorded every 10 s of the following ROI: Chin, perinasal area, nose, periorbital area, and frontal muscles. To evaluate clinical changes, the values of temperature were transformed on a scale from 0 (very low) to 5 (high) in ranges: 0, 0.5, 1, 1.5, 2, 2.5, 3, 3.5, 4, 4.5, and 5 in each ROI (Figure 1c).

*Emotional task.* It was necessary to project two videos; the first one was intended to expose a positive emotional charge (joy) [41], while the other was proposed to expose a negative emotional valence (pain) [42]. The length of each video was 01:31 min. and 01:33 min., respectively. To encourage a facial expression of joy, video 1 showed a child flirting with some adult women by sending them a drink and at the same time winking, eventually, the women’s reaction was to laugh at such behavior. As for negative facial expression, video 2 showed an empathic expression of pain; it showed a woman who helped a soldier, whose main aim was not to return to the war in Afghanistan, pretending that he had suffered an accident that had left him in a wheelchair, and because of that, it was “impossible” for him to feel anything in his legs. After that, his superior officer arrives and hits him with a whip to check if the situation was real. However, when the superior officer hit the soldier’s leg, the person pushing his chair was preparing as if they were about to receive the impact, showing a face of pain.

*FACS* [43]. For the development of the present work, it was essential to use the facial action units of joy (AUs) (AU6 + AU12 + AU25) [43] and the facial action units of pain (AU4 + AU6 + AU7 + AU9 + AU10 + AU20 + AU25 + AU43) [44] according to the Muscle-by-Muscle paradigm. Every measure required the voluntary cooperation of the groups and it was reproduced for at least 10 s [22,45,46].

*Diaphragmatic Recovery/Breathing*. A visual exercise of Heart Math’s biological feedback software emWave^®^ (Pro) was used. This software essentially consists of instructing the subject to visually follow the movement of a ball from the midline upwards to inhale, and expire when it lowers to synchronize the respiratory cycle in order to relax the muscles, including the facials ones, strained by the emotional task.

### 3.3. Procedure

All the measures were conducted on 30 October 2015 at the School of Psychology of the National Autonomous University of Mexico in two contiguous rooms: in the first one, we did the salivary IL-6 and the psychosocial assessment, while in the adjacent room we carried out the thermographic psychophysiological record. Moreover, participants were required to be in a time between 07:30 and 10:00 a.m. At the time of their arriving, they gave us their informed consent and made us know that they met the requirements for taking salivary IL-6, according to the use manual. Once the salivary sample was taken, the psychological scales were answered. This period was also used for the acclimatization of body temperature (15–20 min). Subsequently, they were invited to continue with the psychophysiological evaluation. The scheme of Figure 1b shows the general psychophysiological evaluation procedure. Participants were paired with a two-minute baseline measurement, a five-minute emotional task (involuntary psychophysiological response of video presentation and voluntary psychophysiological response to voluntary facial expression) and, a 2-min recovery period. Due to the nature of the study, the recovery period was assisted by a diaphragmatic breathing exercise with the EM WAVE video. Ethically, this intervention had two purposes; on the one hand, to decrease fatigue associated with emotional facial activity; on the other hand, to weigh the recovery phase and to identify those cases in which the temperature changed according to the activity (emotional task and relaxation). This is to say that not all of these procedures were documented in other studies [47], when it may well contribute to have a more critical perspective.

### 3.4. Statistical Analysis

Statistical analyses were performed with SPSS software, V.21.0 (SPSS, Inc., Chicago, IL, USA).

Friedman’s statistical test of repeated measures was used for thermal psychophysiological evaluation. As for the psychosocial evaluation, the means of each group were compared according to the Shapiro–Wilk normality test; while the IL-6 inflammatory response was compared between the BC survivors group and the clinically healthy group using the Mann–Whitney U-test. We used a multivariate analysis of clusters to differentiate subgroups of participants according to IL-6 levels, and these groups were compared using a simple ANOVA. A linear statistical model of the relationship between the basal temperature level of the nose and the IL-6 was obtained. Finally, two matrices of correlations were obtained: the first one taking into consideration the baseline temperature with the psychosocial aspects; and the other considering the self-report of BPI intensity and its interference with psychosocial measures.

## 4. Results

### 4.1. Inflammatory Response IL-6

The activity of interleukin 6 in saliva was high in both groups; however, it showed no statistically significant differences (*p* = 0.893) between the BC survivors (mean rank: 20.76) and the healthy group (mean rank: 2026). Nevertheless, the analysis of the 3 clusters required the following subdivision (*p* < 0.000): low level of IL-6 (0–5 µg/mL), medium level of IL-6 (5.1–10 µg/mL), and high level of IL-6, 10.1 µg/mL. That being said, and taking into account the control group, it is worth mentioning that 48% of them had a low level, 28% a medium level, and 24% a high level of IL-6. In comparison, the BC survivors showed the following percentages: 46% of them had a low level, 20% a medium level, and 34% a high level of IL-6.

### 4.2. Psychophysiological Assessment through Thermal Infrared Imaging

#### 4.2.1. Baseline

Before the training in the emotional facial expressions, the levels of the initial temperature showed no statistically differences between the survivors of BC and the control group; this is to say that there was almost no fluctuation in the facial ROI of; the chin, the mean rank in patients: 16.2, Md = 4, in control group: 20.91, Md = 4.5 (*p* = 0.202); perinasal area, the mean rank in patients: 16.63, Md = 4, in control group: 20.61, Md = 4.5, (*p* = 0.276); nose, mean rank in patients: 17, Md = 3, in control group: 2036, Md = 4, (*p* = 0.366); periorbital area, mean rank in patients: 19.8, Md = 5, in control group: 1845, Md = 5, (*p* = 0.725); front, mean rank in patients: 19.07, Md = 5, in control group: 18.95, Md = 4.5, (*p* = 0.988); fingers, mean rank in patients: 19.4, Md = 3, in control group: 16.95, Md = 3 (*p* = 0.499).

#### 4.2.2. The Effect of the Emotional Facial Expressions on the Thermal Changes Associated with Facial Temperature in the ROIs

**Figure 2:**
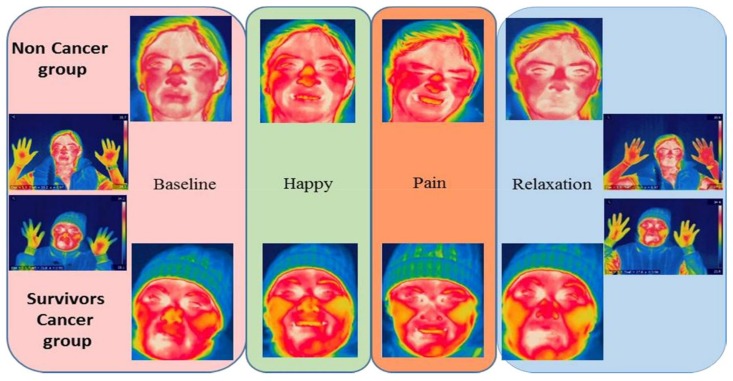
Psychophysiological effect of facial expression. The voluntary facial action had an effect on temperature.

**Figure 3:**
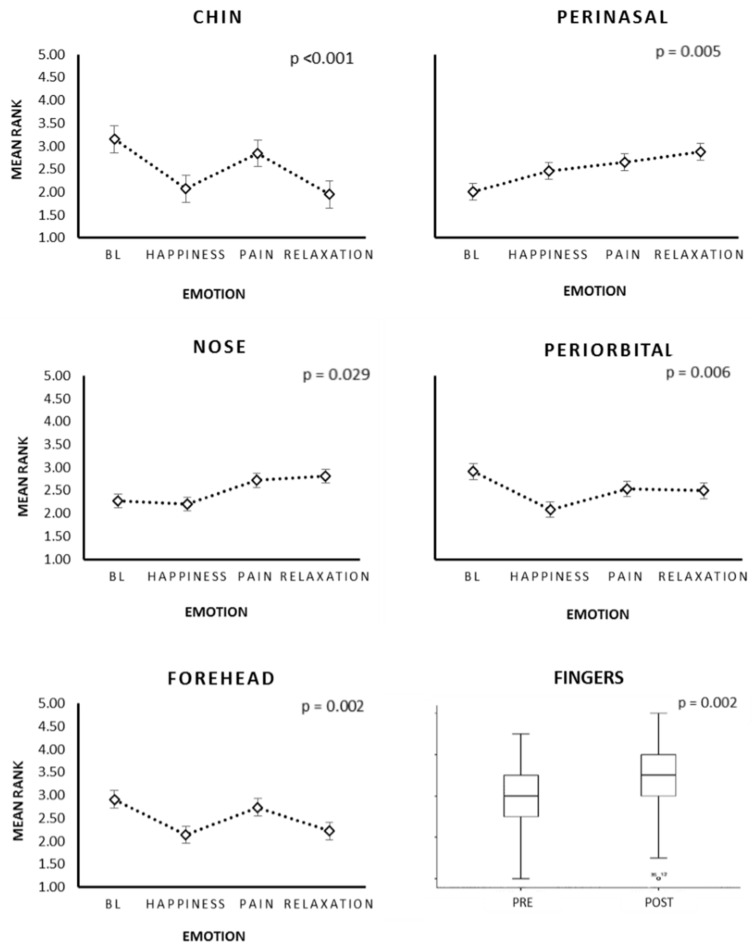
Changes in temperature of each facial region and fingers. The voluntary emotional facial expressions and relaxation had a statistically significant effect. BL = Baseline.

The results obtained indicated that the voluntary facial expression of emotions had effects on the autonomic activity of the peripheral facial temperature. The analysis of the repeated measures demonstrated, in both groups, that there were several changes in the temperature according to the emotional facial expressions. The aforementioned point was clear in the chin (*p* < 0.001), the perinasal area (*p* = 0.005), the nose tip (*p* = 0.029), the periorbital area (*p* = 0.006), and the frontal area (*p* = 0.002) (see Figure 2 and Figure 3).

#### 4.2.3. Thermography: Intra-Group Differences

The facial action units that form the emotional facial expressions changed temperature levels in the ROIs, contributing to noteworthy differences among the control group and the BC survivors. Consequently, the latter showed changes in the following ROIs: chin, perinasal, nose tip, and forehead. However, the chin was the only ROI which showed statistically significant effects in both groups (Table 1).

### 4.3. Relationship between IL-6 and Temperature

**Figure 4:**
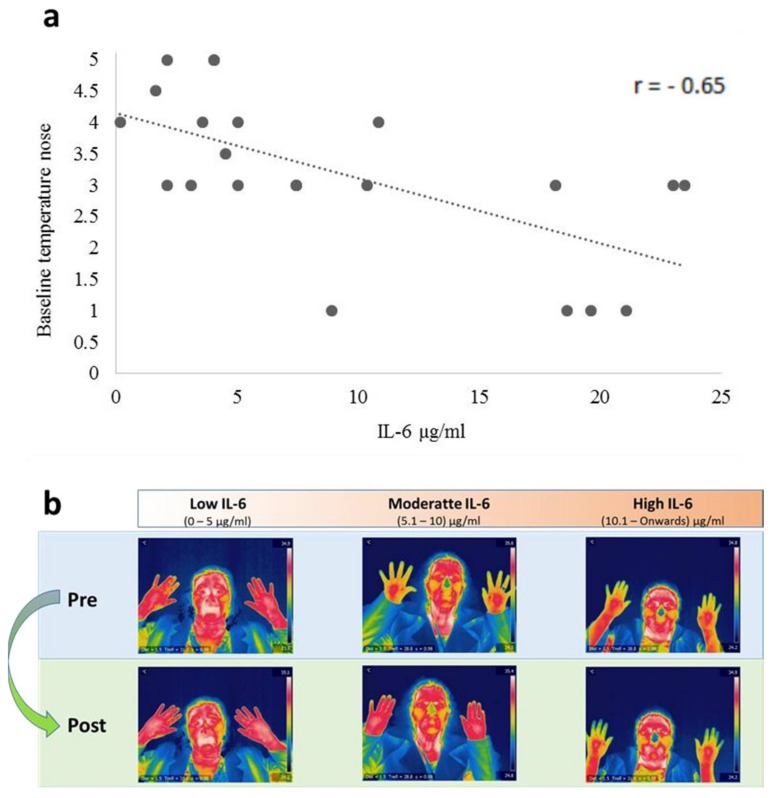
Association between IL-6 and baseline temperature of the nose. The dotted line (**a**) is a negative linear relationship with *p* < 0.001. Participants (**b**) with a low and medium level of IL-6 benefited from facial and relaxation exercises, increasing facial and manual temperature levels, while a high level of IL-6 had no thermal changes.

A negative relationship was found between IL-6 and the levels of basal temperature in the nose tip (*r* = −0.656, *p* < 0.001) and the perinasal area (*r* = −0.559, *p* = 0.007). The explained variance in the temperature of the nose was r^2^ = 0.40 (Figure 4a). Facial exercises and relaxation by diaphragmatic respiration increased the parasympathetic thermal activity in people with low and medium concentrations of IL-6 (Figure 4b).

### 4.4. Psychosocial Assessment of Pain

The results of the psychosocial assessment are shown in Table 2. The pain reported in the group of BC survivors, in the dimension of intensity, was moderate, with an average of 28% relief after the ingestion of drugs for pain. Additionally, we used a multidimensional scale of pain. In this respect, the pain interference was low when it comes to their general activities: affective state, social and interpersonal relationships, daily life activities, sleep, and enjoyment of life. However, pain did interfere with walking ability. The NAS of SF-MPQ indicated a level of pain under the general average, with predominance in its sensory dimension regarding the affective aspect. Nonetheless, when comparing both groups, only statistically significant differences were found (*p* < 0.05) in the scores of post-traumatic stress symptoms and social isolation (BC group < group clinically healthy); however, the score of alexithymia was higher (*p* < 0.01) in the group of BC survivors and, in particular, in the sub-scale *Difficulty in identifying emotions and feelings.*

### 4.5. Relationship between IL-6 and Psychosocial Characteristics

It was necessary to identify the relationships between the initial temperature of each ROI and psychosocial characteristics. The results are displayed in Table 3. The level of IL-6 in the BC group was inversely associated with the mood (*r*= −0.593, *p* = 0.02), walking (*r* = −0.544, *p* = 0.036), and the ability to work (*r* = −0.583, *p* = 0.022).

### 4.6. The Relationship of Psychosocial Characteristics

We evaluated the relationships of the psychosocial aspects. In fact, multiple correlations were found, not only between the pain and its interference, but also in the affective aspect (Table 4).

## 5. Discussion

The initial analysis of IL-6 data showed no difference between the group of BC survivors and the control group, as in other investigations [48]. However, the analysis of IL-6 allowed us to distinguish three sub-groups of participants, according to their low, moderate, or high level. Despite the fact that no differences were found in the psychological measures between these three sub-groups, there are statistical differences in the basal nasal temperature. These results indicated an inverse relationship between IL-6 and the basal nasal temperature level. Actually, sixty-six percent of the participants, from both the BC group and control group with a low and a moderate level of IL-6, were able to regulate their facial temperature in the following areas: the tip of the nose, the maxillary area, and the fingers, all of this by exposing the patients to specific videos and the realization of facial exercises. While the remainder (34%) of the sample with high levels of IL-6 concentration could not regulate its peripheral temperature. This permitted us to identify people with thermal plasticity based on their level of inflammation. In fact, thermal plasticity could be a clinical biomarker of the particular needs of each patient in the biological dimension of their biopsychosocial profile. In patients who did not present changes after the facial or the relaxation exercises, it has been concluded that a pain specialist should treat their higher level of inflammatory response.

At the beginning of the study, the dynamic evaluation of the facial peripheral temperature showed that there were no differences between the groups. This suggests that those changes in temperature can be attributed solely to the emotional task. The pre/post results indicated that temperature changes in different facial regions and fingers had a distinct activity: while temperature increases in the fingers, the perinasal area, and the tip of the nose presumably due to the predominantly parasympathetic activity, a decrease in temperature occurred in the chin, which is consistent with the relaxation of the mandible and tassel muscle. This is concurrent with the participation of AUs that distinguish between one emotion and the others: emotional facial expression is distinguishable in accordance with the action unit that contracts the muscles around the mouth, observable through the AUs and their inactivity during relaxation of the blood flow, which generates, consequently, a lower temperature. Because some AUs are more complicated to perform [49], the same autonomic effect is not achieved in all the participants. As a result, while some body regions increase their temperature, others experience a decrease, according to the facial expression and the blood flow required by each expression.

The initial temperature of each ROI was related to psychosocial scales. Finger temperature was inversely connected with behavioral interference and intensity of pain, as well as social isolation. This is consistent with a sympathetic domain of the activity [50].

In addition, the breast cancer survivors particularly showed significant psychosocial characteristics, including a low level of pain and low interference with their daily lives, which promote autonomic regulation. Furthermore, it was observed that they experienced a better social involvement compared to the clinically healthy group, which is consistent with their low level of pain and their low depression score [14]. In fact, the only clinical characteristic that we found for their psychological care was alexithymia, which was higher in comparison with the control group. This is of special clinical interest because alexithymia was the only psychological variable that was positively related to a general level of pain, coinciding with the findings reported by other researchers [25,37]. This suggests that, for patients with a high level of pain, the design of a treatment based on a brief training in recognition and emotional facial expression would decrease their score of alexithymia, and hence of pain. Also, while facial expression could lead to changes in the autonomic activity, which is associated with the inflammatory response; emotional facial recognition may well decrease their alexithymia scores when it comes to identifying emotions.

Finally, the biopsychosocial profile of pain in this group of cancer survivors, unlike the biopsychosocial predictors of endometrial surgery pain [3], showed the following characteristics: level of pain ranging from low to moderate, low behavioral interference, no anxiety, absence of depression, a low mood of anger, medium control of anger (external and internal), no post-traumatic stress, low social isolation, moderate alexithymia (in particular as for the recognition of emotions and feelings), medium level of cognitive reevaluation, and emotional suppression. The usefulness of the training of this group has provided social support, and thus it facilitates the interaction between its members, the doctors, and the specialists, coinciding with Reinertsen’s work [51]. In addition, it is worth noting that social support has been documented as a psychosocial variable capable of reducing stress [9], which undoubtedly promotes an adaptive state of disease behavior [14]. In this regard, it should be observed that facial expressions are useful for emotional communication because it enables and optimizes social interactions.

## 6. Conclusions

The sum and interaction, not only of the psychobiological components (inflammatory response and autonomic activity), but also of the psychosocial ones, made it possible to characterize this group of female survivors of BC. Nevertheless, it is crucial to continue treating this group with all the possible measures to assess the usefulness of this biopsychosocial evaluation. The psychophysiological evaluation of facial thermoregulation adds evidence about the autonomic changes that promote voluntary or learned facial expression. However, what is missing is the quantification of the temperature that could permit us to obtain the confidence parameters of the normative ranges of a specific facial movement or its interaction with others. Psychosocial aspects have been documented in this clinical research, although it does not mean that they are the only relevant affective variables. Actually, it is necessary to determine the influence of other cognitive aspects, such as the bias in attention, that allow us to more efficiently regulate the affective and psychophysiological processes on pain; but, in other similar studies [51], the diaphragmatic respiration reduced psychophysiological measures in people with low and medium concentrations of IL-6. Therefore, it is necessary to research psychological strategies in persons with high concentrations of IL-6 to reduce their autonomic activity.

## Figures and Tables

**Table 1:** Means ranks of each ROI per emotion in the Ca—Mama group and healthy group.

ROI	CA—Mama Group						Healthy Group					
	BL	Ha	Pa	Rlx	X^2^	p	BL	Ha	Pa	Rlx	X^2^	p
Chin	3.03	2	3.1	1.87	15.87	0.002 **	3.22	2.06	2.58	2.14	13.87	0.003 **
Perinasal	1.9	2.3	2.93	2.97	8.40	0.038 *	2.14	2.5	2.44	2.92	5.33	0.149
Nose	1.93	2.2	3.1	2.77	10.15	0.017 *	2.56	2.19	2.39	2.86	3.63	0.304
Periorbital	2.9	2.17	2.57	2.37	4.88	0.18	2.78	2.08	2.58	2.56	4.26	0.235
Forehead	2.8	1.93	2.9	2.37	9.93	0.019 *	2.92	2.19	2.64	2.25	5.13	0.162

The Friedman test of measures repeated (*X*^2^) shown differences intragroup. BL = Baseline, Ha = Happiness, Pa = Pain, Rlx = Relaxation. * *p* < 0.05, ** *p* < 0.01.

**Table 2:** Psychosocial assessment of pain.

Psychometric Outcomes	CA-Mama Group	Health Group	*p*-Value
	Mean (SD)	Mean (SD)	
Pain (BPI)			
*Maximum*	4.73 (3.05)	3.57 (3.10)	0.418
*Minimum*	2.73 (2.12)	2.14 (2.79)	0.588
*Mean*	4.13 (2.77)	3.42 (2.57)	0.577
*Current*	2.53 (2.29)	2.28 (3.14)	0.836
*Relief (percentage)*	28 (37.64)	24.28 (38.23)	0.832
Interference (BPI)			
*General Activity*	3.20 (3.07)	2.14 (3.67)	0.488
*Affective State*	3.33 (3.79)	1.85 (3.07)	0.380
*Walking*	3.91 (1.98)	0.71 (1.88)	0.017 *
*Working*	2.26 (3.55)	1.42 (3.77)	0.281
*Relationship*	2.86 (3.66)	1.71 (3.72)	0.502
*Sleep*	2.73 (3.17)	3.28 (4.27)	0.737
*Enjoy*	2.53 (4.01)	1.85 (3.18)	0.767
Pain’s Qualities (SF-MPQ)			
*Intensity* (NAS)	1.33 (1.17)	0.727 (0.786)	0.152
*Sensory*	5.00 (3.9)	2.54 (3.07)	0.102
*Affective*	0.600 (1.35)	0.000 (0.000)	0.108
Anxiety (HADS-A)	5.67 (3.13)	6.22 (3.93)	0.661
Depression (HADS-D)	3.00 (3.16)	3.94 (3.65)	0.464
Anger (STAXI-2)			
*State*	2.67 (2.41)	4.50 (4.09)	0.137
*Temperament*	3.13 (2.10)	3.82 (4.24)	0.544
*External Control*	10.47 (4.92)	11.47 (4.07)	0.449
*Internal Control*	13.07 (5.63)	10.67 (5.33)	0.219
Posttraumatic Stress (PC-PTSD)	0.67 (0.90)	1.55 (1.036)	0.036 *
Loneliness (L-UCLA)	30.40 (10.88)	36.78 (9.22)	0.044 *
Alexithymia (TAS)			
*Total*	26.20 (12.68)	13.23 (8.66)	0.008 **
*Identification*	14.50 (7.44)	6.23 (4.74)	0.004 **
*Expression*	11.70 (6.16)	7.00 (5.38)	0.065
Emotional Regulation (EQR)			
*Cognitive Reappraisal*	27.10 (6.33)	29.92 (6.13)	0.293
*Suppression*	14.10 (6.38)	11.00 (4.47)	0.376

The means and standard deviations (SD) of the psychometric scores in each group were presented and compared with a student’s *t*-test as a function of the normality of the Shapiro–Wilk test. * *p* < 0.05, ** *p* < 0.01.

**Table 3:** Relationship between initial temperature of ROIs and psychosocial aspects.

RIO—Psychosocial Measure	Coefficient of Correlation	
Temp. Fingers—Temp. Nose	0.450	**
—Interference General (BPI)	−0.390	*
—Interference Affective (BPI)	−0.517	*
—Interference Relationship (BPI)	−0.437	*
—Interference Sleep (BPI)	−0.600	***
—Interference Enjoy (BPI)	−0.665	***
—Pain (NAS of SF-MPQ)	−0.584	**
—Loneliness (L-UCLA)	−0.487	**
Temp. Chin—Temp. Maxillofacial	0.483	**
—Temp. Nose	0.459	**
—Internal Control of Anger (STAXI-2)	0.383	*
—Alexithymia (TAS)	−0.446	*
—Supression (EQR)	−0.481	*
Temp. Maxillary—Temp. Nose	0.821	***
—Interference Sleep (BPI)	−0.520	**
—Interference Enjoy (BPI)	−0.461	**
—Pain (NAS of SF-MPQ)	−0.504	**
Temp. Nose—Interference Sleep (BPI)	−0.443	*
—Pain (NAS of SF-MPQ)	−0.413	*
Temp. Periorbital—Temp. Forehead	0.469	**
—Reappraisal (EQR)	−0.485	*

Statistically significant correlations (*p* < 0.05). * = *p* < 0.05, ** = *p* < 0.01, *** = *p* < 0.001.

**Table 4:** Psychosocial matrix correlations.

Measure	Correlation of Coefficient		Measure	Correlation of Coefficient	
***Pain Maximum***			***Anxiety***		
Minimum	0.813	***	Depression	0.687	***
Mean	0.724	***	Interference Enjoy	0.496	**
Current	0.742	***	PTSD	0.737	***
Interference General Activity	0.651	***	Loneliness	0.462	**
Interference Affective State	0.614	***	State of anger	0.478	**
Interference Walking	0.708	***	Temperament anger	0.446	**
Interference Working	0.655	***	Internal control anger	−0.549	***
NAS of Pain	0.551	**	Alexithymia	0.578	**
Sensory Pain	0.508	**	*Alexithymia ID*	0.556	**
***Pain Minimum***		-	*Alexithymia Expression*	0.496	*
Mean	0.795	***	Suppression	0.417	*
Current	0.943	***	***Depression***		
Relief	0.535	**	Interference Enjoy	0.454	*
Interference Affective State	0.632	***	PTSD	0.750	***
Interference Walking	0.652	***	Loneliness	0.342	*
Interference Working	0.692	***	State of anger	0.453	**
Interference Slipping	0.507	**	Temperament anger	0.345	*
NAS of Pain	0.672	***	Internal control anger	−0.611	***
Sensory Pain	0.590	***	***PTSD***		
Internal control of Anger	0.438	*	Loneliness	0.510	**
***Pain Current***			State of anger	0.768	***
Relief	0.614	***	Anger State		
Interference General Activity	0.715	***	Loneliness	0.392	*
Interference Affective State	0.659	***	Internal Control anger	−0.455	**
Interference Walking	0.580	**	External Control Anger		
Interference Working	0.660	***	Internal Control anger	0.482	**
Interference sleeping	0.681	***	Suppression	−0.462	*
Interference Enjoy	0.430	*	***Internal Control Anger***		
NAS of Pain	0.686	***	Loneliness	−0.390	*
Sensory Pain	0.689	**	Alexithymia	−0.423	*
***Relief***			-Alexithymia Expression	−0.474	*
Interference sleeping	0.524	**	Suppression	−0.666	***
***Interference General Activity***			***Alexithymia***		
Interference Affective State	0.875	***	Interference General Activity	0.662	*
Interference Walking	0.778	***	Suppression	0.501	*
Interference Working	0.832	***	*Alexithymia ID*		
Interference sleeping	0.464	*	Interference General Activity	0.784	**
Interference Enjoy	0.570	**	Interference Affective State	0.635	*
NAS of Pain	0.711	***	NAS of Pain	0.652	*
Sensory Pain	0.626	***	*-Alexithymia Expression*	0.732	***
***Interference Affective State***			***Alexithymia Expression***		
Interference Walking	0.816	***	Interference General Activity	0.611	*
Interference Working	0.894	***	Internal Control anger	−0.474	*
Interference in Relationship	0.481	*	Suppression	0.566	**
Interference sleeping	0.596	*	***Suppression***		
Interference Enjoy	0.693		Sensory Pain	0.650	**
NAS of Pain	0.609	***	Affective Pain	0.748	**
Sensory Pain	0.569	**	***Loneliness***		
***Interference Working***			Interference Affective State	0.392	*
Interference sleeping	0.461	*	Interference sleeping	0.479	*
Interference Enjoy	0.545	**	Interference Enjoy	0.476	*
NAS of Pain	0.609	***	Alexithymia Expression	0.416	*
Sensory Pain	0.579	**			

Statistically significant correlations (*p* < 0.05). NAS = Numerical Analogue Scale of Pain; Alexithymia ID = Alexithymia in Identification of emotions. * = *p* < 0.05, ** = *p* < 0.01, *** = *p* < 0.001.

## References

[1] Schreiber K.L., Kehlet H., Belfer I., Edwards R.R. (2014). Predicting, preventing and managing persistent pain after breast cancer surgery: The importance of psychosocial factors. Pain Manag..

[2] Nijs J., Leysen L., Adriaenssens N., Aguilar Ferrandiz M.E., Devoogdt N., Tassenoy A., Ickmans K., Goubert D., van Wilgen C.P., Wijma A.J. (2016). Pain following cancer treatment: Guidelines for the clinical classification of predominant neuropathic, nociceptive and central sensitization pain. Acta Oncol..

[3] Honerlaw K.R., Rumble M.E., Rose S.L., Coe C.L., Costanzo E.S. (2016). Biopsychosocial predictors of pain among women recovering from surgery for endometrial cancer. Gynecol. Oncol..

[4] Bredal I.S., Smeby N.A., Ottesen S., Warncke T., Schlichting E. (2014). Chronic Pain in Breast Cancer Survivors: Comparison of Psychosocial, Surgical, and Medical Characteristics between Survivors with and without Pain. J. Pain Symptom Manag..

[5] Novy D.M., Aigner C.J. (2014). The biopsychosocial model in cancer pain. Curr. Opin. Support. Palliat. Care.

[6] Belfer I., Schreiber K.L., Shaffer J.R., Shnol H., Blane K., Morando A., Englert D., Greco C., Brufsky A., Ahrendt G. (2013). Persistent Postmastectomy Pain in Breast Cancer Survivors: Analysis of Clinical, Demographic, and Psychosocial Factors. J. Pain.

[7] Yanez B., Thompson E.H., Stanton A.L. (2011). Quality of life among Latina breast cancer patients: A systematic review of the literature. J. Cancer Surviv..

[8] Porro M., Andrés M., Rodríguez S. (2012). Regulación Emocional y Cáncer: Utilización Diferencial de la Expresión y Supresión Emocional en Pacientes Oncológicos. Avances En Psicología Latinoamericana. http://revistas.urosaROI.edu.co/index.php/apl/article/view/1969/1967.

[9] Wittig R.M., Crockford C., Weltring A., Langergraber K., Deschner T., Zuberbühler K. (2016). Social support reduces stress hormone levels in wild chimpanzees across stressful events and everyday affiliations. Nat. Commun..

[10] DeVon H.A., Piano M.R., Rosenfeld A.G., Hoppensteadt D.A. (2014). The Association of Pain with Protein Inflammatory Biomarkers. Nurs. Res..

[11] Bäckryd E. (2015). Pain in the Blood? Envisioning Mechanism-Based Diagnoses and Biomarkers in Clinical Pain Medicine. Diagnostics.

[12] Lasselin J., Elsenbruch S., Lekander M., Axelsson J., Karshikoff B., Grigoleit J., Engler H., Schedlowski M., Benson S. (2016). Mood disturbance during experimental endotoxemia: Predictors of state anxiety as a psychological component of sickness behavior. Brain Behav. Immun..

[13] Shattuck E.C., Muehlenbein M.P. (2016). Towards an integrative picture of human sickness behavior. Brain Behav. Immun..

[14] Hughes S., Jaremka L.M., Alfano C.M., Glaser R., Povoski S.P., Lipari A.M., Agnese D.M., Farrar W.B., Yee L.D., Carson W.E. (2014). Social support predicts inflammation, pain, and depressive symptoms: Longitudinal relationships among breast cancer survivors. Psychoneuroendocrinology.

[15] Fernández-Cuevas I., Marins J.C.B., Lastras J.A., Carmona P.M.G., Cano S.P., García-Concepción M.Á., Sillero-Quintana M. (2015). Classification of factors influencing the use of infrared thermography in humans: A review. Infrared Phys. Technol..

[16] McIntosh N.D., Zajonc R.B., Vig P.S., Emerick S.W. (1997). Facial Movement, Breathing, Temperature, and Affect: Implications of the Vascular Theory of Emotional Efference. Cognit. Emot..

[17] Rustemeyer J., Radtke J., Bremerich A. (2007). Thermography and thermoregulation of the face. Head Face Med..

[18] Ioannou S., Gallese V., Merla A. (2014). Thermal infrared imaging in psychophysiology: Potentialities and limits. Psychophysiology.

[19] Khan M.M., Ingleby M., Ward R.D. (2006). Automated Facial Expression Classification and affect interpretation using infrared measurement of facial skin temperature variations. ACM Trans. Auton. Adapt. Syst..

[20] Wang S., Liu Z., Lv S., Lv Y., Wu G., Peng P., Chen F., Wang X. (2010). A Natural Visible and Infrared Facial Expression Database for Expression Recognition and Emotion Inference. IEEE Trans. Multimed..

[21] Jarlier S., Grandjean D., Delplanque S., N’Diaye K., Cayeux I., Velazco M., Sander D., Vuilleuimer P., Sherer K. (2011). Thermal Analysis of Facial Muscles Contractions. IEEE Trans. Affect. Comput..

[22] Levenson R.W., Ekman P., Friesen W.V. (1990). Voluntary Facial Action Generates Emotion-Specific Autonomic Nervous System Activity. Psychophysiology.

[23] Kraft T.L., Pressman S.D. (2012). Grin and bear it: The influence of manipulated facial expression on the stress response. Psychol. Sci..

[24] Price T.F., Harmon-Jones E. (2015). Embodied emotion: The influence of manipulated facial and bodily states on emotive responses. Wiley Interdiscip. Rev. Cognit. Sci..

[25] Baudic S., Jayr C., Albi-Feldzer A., Fermanian J., Masselin-Dubois A., Bouhassira D., Attal N. (2016). Effect of Alexithymia and Emotional Repression on Postsurgical Pain in Women with Breast Cancer: A Prospective Longitudinal 12-Month Study. J. Pain.

[26] Melzack R. (1987). The short-form McGill pain questionnaire. Pain.

[27] Escalante A., Lichtenstein M., Ríos N., Hazuda H. (1996). Measuring chronic rheumatic Pain in Mexican Americans: Cross-cultural adaptation of the McGill Pain Questionnaire. J. Clin. Epidemiol..

[28] Hawker G.A., Mian S., Kendzerska T., French M. (2011). Measures of adult pain: Visual Analog Scale for Pain (VAS Pain), Numeric Rating Scale for Pain (NRS Pain), McGill Pain Questionnaire (MPQ), Short-Form McGill Pain Questionnaire (SF-MPQ), Chronic Pain Grade Scale (CPGS), Short Form-36 Bodily Pain Scale (SF). Arthritis Care Res..

[29] Dudgeon D., Raubertas R.F., Rosenthal S.N. (1993). The short-form McGill Pain Questionnaire in chronic cancer pain. J. Pain Symptom Manag..

[30] Cleeland C. (2009). The Brief Pain Inventory User Guide. http://sosmanuals.com/manuals/cae48184b7bae3b1d85105ea85375b60.pdf.

[31] Galindo O., Benjet C., Juárez F., Rojas E., Riveros A., Aguilar J., Á lvarez M., Alvarado S. (2015). Psychometric properties of the Hospital Anxiety and Depression Scale (HADS) in a Mexican population of cancer patients. Salud Ment..

[32] Prins A., Ouimette P., Kimerling R., Cameron R., Hugelshofer D., Shaw-Hegwer J., Thrailkill A., Gusman F., Sheikh J. (2003). The Primary Care PTSD Screen (PC-PTSD): Development and operating characteristics (PDF). Prim. Care Psychiatry.

[33] Tauben D. (2012). Chronic Pain Management: Measurement-Based Step Care Solutions—IASP. http://www.iasp-pain.org/PublicationsNews/NewsletterIssue.aspx?ItemNumber=2064.

[34] Oliva F., Hernández M., Calleja N. (2010). Validation of the Mexican Version of the State-Trait Ander Expression Inventory (STAXI-2). Acta Colombiana de Psicología. http://www.redalyc.org/pdf/798/79819279010.pdf.

[35] Taylor G.J., Ryan D., Bagby R.M. (1985). Toward the Development of a New Self-Report Alexithymia Scale. Psychother. Psychosom..

[36] Durán W. (2007). Validación de la Escala de Alexitimia de Toronto (TAS-20). http://catarina.udlap.mx/u_dl_a/tales/documentos/lps/weisel_d_m/indice.html.

[37] Shibata M., Ninomiya T., Jensen M., Anno K., Yonmoto K., Makino S., Iwaki R., Yashimiro K., Yoshida T., Imada Y. (2014). Alexithymia Is Associated with Greater Risk of Chronic Pain and Negative Affect and with Lower Life Satisfaction in a General Population: The Hisayama Study. PLoS ONE.

[38] Cabello R., Salguero J., Fernández-Berrocal P., Gross J. (2013). A Spanish Adaptation of the Emotion Regulation Questionnaire. Eur. J. Psychol. Assess..

[39] Russell D. (1996). UCLA Loneliness Scale (Version 3): Reliability, Validity, and Factor Structure. J. Personal. Assess..

[40] Sillero M., Fernández I., Arnaiz J., Bouzas J. (2015). Protocol for thermographic assessment in humans. PreCongress XIII EAT Congress Course on “Medical Applications of Human Thermography”.

[41] (2011). Military Torture Interrogation Prank. https://www.youtube.com/watch?v=SF2g6oxIfQ.

[42] (2011). Cute Kid Gets Caught Buying Drinks for the Ladies. https://www.youtube.com/watch?v=J_9UV3fiW0E.

[43] Domínguez Sánchez F., Fernández E., García B., Jiménez M., Martín M., Domínguez F. (2010). La Alegria, la Tristeza y la Ira. Psicología de la Emoció N.

[44] Chambers C., Mogil J. (2015). Ontogeny and phylogeny of facial expression of pain. Pain.

[45] Cohn J., Ambadar Z., Ekman P., Cohan J., Allen J. (2007). Observer-Based Measurement of Facial Expression with the Facial Action Coding System. Handbook of Emotion Elicitation and Assessment.

[46] Boiten F. (1996). Autonomic response patterns during voluntary facial action. Psychophysiology.

[47] Tyler W.J., Boasso A.M., Mortimore H.M., Silva R.S., Charlesworth J.D., Marlin M.A., Aebersold K., Aven L., Wetmore D.Z., Pal S.K. (2015). Transdermal neuromodulation of noradrenergic activity suppresses psychophysiological and biochemical stress responses in humans. Sci. Rep..

[48] Knüpfer H., Preiß R. (2007). Significance of interleukin-6 (IL-6) in breast cancer (review). Breast Cancer Res. Treat..

[49] Gosselin P., Perron M., Beaupré M. (2010). The voluntary control of facial action units in adults. Emotion.

[50] Kreibig S.D. (2010). Autonomic nervous system activity in emotion: A review. Biol. Psychol..

[51] Jafari H., Courtois I., Van den Bergh O., Viaeyen J., Van Diest I. (2017). Pain and respiration: A systematic review. Pain.

